# Antibacterial, Antifungal, Antiviral, and Anthelmintic Activities of Medicinal Plants of Nepal Selected Based on Ethnobotanical Evidence

**DOI:** 10.1155/2020/1043471

**Published:** 2020-04-22

**Authors:** Bishnu Joshi, Sujogya Kumar Panda, Ramin Saleh Jouneghani, Maoxuan Liu, Niranjan Parajuli, Pieter Leyssen, Johan Neyts, Walter Luyten

**Affiliations:** ^1^Department of Pharmaceutical and Pharmacological Sciences, KU Leuven, Herestraat 49, Box 921, 3000 Leuven, Belgium; ^2^Central Department of Biotechnology, Tribhuvan University, Kirtipur, 9503 Kathmandu, Nepal; ^3^Department of Biology, KU Leuven, Naamsestraat 59, 3000 Leuven, Belgium; ^4^Central Department of Chemistry, Tribhuvan University, Kirtipur, Kathmandu, Nepal; ^5^Rega Institute for Medical Research, Laboratory of Virology and Chemotherapy, KU Leuven, Herestraat 49, 3000 Leuven, Belgium

## Abstract

**Background:**

Infections by microbes (viruses, bacteria, and fungi) and parasites can cause serious diseases in both humans and animals. Heavy use of antimicrobials has created selective pressure and caused resistance to currently available antibiotics, hence the need for finding new and better antibiotics. Natural products, especially from plants, are known for their medicinal properties, including antimicrobial and anthelmintic activities. Geoclimatic variation, together with diversity in ethnomedicinal traditions, has made the Himalayas of Nepal an invaluable repository of traditional medicinal plants. We studied antiviral, antibacterial, antifungal, and anthelmintic activities of medicinal plants, selected based upon ethnobotanical evidence.

**Methods:**

Ethanolic and methanolic extracts were tested (1) on a panel of microbes: two Gram-positive bacteria (*Staphylococcus aureus* and *Listeria innocua*), four Gram-negative bacteria (*Escherichia coli*, *Pseudomonas aeruginosa*, *Salmonella enterica*, and *Shigella sonnei*), and one fungal species: *Candida albicans*; (2) against three different viruses: yellow fever, chikungunya, and enterovirus; and (3) on the nematode *Caenorhabditis elegans*. Also, cytotoxicity was assessed on human hepatoma (Huh), rhabdosarcoma (RD), and Vero (VC) cell lines.

**Results:**

Of 18 plants studied, *Ampelocissus tomentosa* and *Aleuritopteris anceps* inhibited *S. aureus* (MIC 35 *μ*g/mL and 649 *μ*g/mL, respectively) and *Pseudomonas aeruginosa* (MIC 15 *μ*g/mL and 38 *μ*g/mL, respectively). *Rhododendron arboreum* and *Adhatoda vasica* inhibited *S. enterica* (MIC 285 *μ*g/mL and 326 *μ*g/mL, respectively). *Kalanchoe pinnata, Ampelocissus tomentosa*, and *Paris polyphylla* were active against chikungunya virus, and *Clerodendrum serratum* was active against yellow fever virus (EC_50_ 15.9 *μ*g/mL); *Terminalia chebula* was active against enterovirus (EC_50_ 10.6 *μ*g/mL). *Ampelocissus tomentosa, Boenninghausenia albiflora, Dichrocephala integrifolia*, and *Kalanchoe pinnata* significantly reduced *C. elegans* motility, comparable to levamisole.

**Conclusions:**

In countries like Nepal, with a high burden of infectious and parasitic diseases, and a current health system unable to combat the burden of diseases, evaluation of local plants as a treatment or potential source of drugs can help expand treatment options. Screening plants against a broad range of pathogens (bacteria, viruses, fungi, and parasites) will support bioprospecting in Nepal, which may eventually lead to new drug development.

## 1. Background

Infectious diseases are the major cause of morbidity and mortality and thus a serious public health problem in developing countries. Despite the arsenal of antibiotics available, the situation is worsening due to emerging drug resistance. Antimicrobial resistance in a hospital setting is a major issue these days due to the extensive use or misuse of these drugs. Hence, there is an urgent need to find new molecules to treat infectious diseases [1, 2]. An attractive strategy for finding such molecules would be to test plants used to treat infectious diseases in traditional systems of healing, since medicinal plants have been a source of many pharmaceutical drugs for a range of diseases, including viral, bacterial, fungal, and protozoal infections, as well as for cancer [3, 4]. Over the past four decades, natural products were the direct or indirect source of approximately 50% of newly approved drugs [5, 6]. Plant products are often seen as natural, readily available, and having few side effects. Besides the popularity of herbal drugs, the use of herbal cosmetics and nutraceuticals has been increasing worldwide [7]. However, screening of different plant parts against a wide range of pathogens is cumbersome and requires significant resources. However, plant selection based on ethnobotany and traditional practices such as Ayurveda, Yunani, Siddha, Traditional Chinese Medicine, and Japanese Kampo medicine increases the probability of finding bioactive molecules that may be subsequently developed clinically [8, 9].

Asia represents one of the important centers of knowledge about plant species for the treatment of various ailments. Within this region, Nepal is rich in (medicinal) plant biodiversity, and linking this with the indigenous knowledge on medicinal and aromatic plants provides an attractive approach for the development of novel (e.g., anti-infective) drugs. “Medicinal plants have a long tradition, but ethnopharmacology as a well-defined field of research has a relatively short history, dating back about 50 years only” [10, 11]. Ethnopharmacological selection combined with testing on a range of pathogens (viruses, bacteria, fungi, and protozoa) is a more efficient way of identifying new bioactive molecules than testing randomly selected plants. Therefore, we tested the antiviral, antibacterial, antifungal, and anthelmintic activities of 18 medicinal plants of Nepal, selected based on ethnobotanical evidence for their use in infectious disease.

## 2. Methods

### 2.1. Plant Materials

Eighteen plant species were collected for evaluation of their antibacterial, antifungal, antiviral, and anthelmintic activity based on their uses in the scientific literature and indigenous knowledge. Bark, leaves, fruit, or roots of the selected plants (Table 1) were separately collected from different regions in Nepal from December 2013 to April 2014. The rules for plant collection and identification were followed according to the Department of Plant Resources, Ministry of Forest and Environment, Government of Nepal. All the collected plant materials were identified by professional taxonomists at the Department of Plant Resources, and voucher specimens [63] were deposited in the National Herbarium and Plant Laboratories, Department of Plant Resources, Ministry of Forest and Environment, Government of Nepal. The information on these plants with their official taxonomic name (according to the plant list: http://www.theplantlist.org), indigenous use, used part(s), literature availability, and voucher number is shown in Table 1. Among the collected plants, none are endangered or rare species.

### 2.2. Reagents

Ethanol, methanol, DMSO, growth media (components), ciprofloxacin hydrochloride, and miconazole were obtained from Sigma-Aldrich (Belgium). Water was obtained from a Milli-Q® Integral Water Purification System for ultrapure water (Millipore).

### 2.3. Extraction

The collected plant materials were washed thoroughly with tap water and shade-dried at ambient temperature. Dried samples were ground to a powder with an electric blender and subjected to Soxhlet extraction using polar solvents (ethanol and methanol). The extracts were evaporated on a rotary evaporator under reduced pressure till a solid mass was obtained. All the extracts were kept in sealed vials, labelled with appropriate code, and transported by airmail from Nepal to the KU Leuven, Belgium, for integrated *in vitro* screening. The dried extracts were kept at 4°C until analysis.

### 2.4. Microbial Strains

The *in vitro* antibacterial activity of the crude extract was evaluated using a panel of pathogenic strains including Gram-positive bacteria: *Staphylococcus aureus* (ATCC 6538, Rosenbach) and *Listeria innocua* (LMG 11387), Gram-negative bacteria: *Escherichia coli* (DH10B), *Pseudomonas aeruginosa* (PAO1), *Salmonella enterica* subsp. enterica (ATCC 13076), and *Shigella sonnei* (LMG 10473), and one fungal strain: *Candida albicans* (SC5314). The (frozen) storage, maintenance, and preparation of working culture suspensions were carried out following established procedures described in our previous studies [64].

### 2.5. Antimicrobial Assay

Antimicrobial activity was assessed as described in our previous study [64, 65]. In brief, two-fold serial dilutions of the agents were prepared in DMSO using sterile 96-well microdilution plates with flat-bottom wells. Antibiotics ciprofloxacin and miconazole were used as positive control for bacteria and fungi, respectively. For bacteria, 5% DMSO was used as the solvent control for all the experiments, while for *C. albicans,* 2% DMSO was used. The MIC was calculated by IC_50_/IC_90_ Laboratory Excel Calculation Tools and expressed as IC_50_ as developed at the University of Antwerp (http://www.leishrisk.net/leishrisk/userfiles/file/kaladrugr/sops/lab/labsop18_ic50tool.pdf).

### 2.6. Anthelmintic Test

The anthelmintic assay was carried out in a 96-well microplate with flat-bottom wells in a WMicrotracker (Phylumtech, Argentina) apparatus, as described in our previous study [66]. The movement of worms in each well was measured every 30 minutes for 16 h at 20°C and recorded by the WMicrotracker. The percentage of the average movement within 16 hours of worms exposed to samples, compared to a DMSO control, was used to calculate the relative anthelmintic activity. Levamisole (50 *μ*M) was used on every plate as a positive control, and DMSO was used as solvent control.

### 2.7. Antiviral Test

Antiviral activity was tested as described earlier [65, 67]. The final, maximal DMSO concentration in the assay wells with the highest sample input (1%) was well tolerated by the cells. Rupintrivir was used as a positive control. All data were expressed as EC_50_, CC_50_, and SI. For cytotoxicity assays, plant extracts (only those that showed antiviral activity) were evaluated by the MTS method. In brief, the same experimental setup was used as for the antiviral assay; except for cytotoxicity determination, uninfected cultures were incubated with a serial dilution of plant extract for three days at 37°C. “The cytotoxic concentration was calculated as CC_50_ or the concentration of plant extracts required to reduce cell proliferation by 50% relative to the number of cells in the solvent-treated controls. The formula used to calculate cytotoxic activity was % CPE = [OD_cc_ − OD plant extract]/OD_cc_, where OD_cc_ corresponds to the optical density of the uninfected and untreated cell cultures, and OD plant extract corresponds to the OD of uninfected cultures, treated with the extract. In addition, the selectivity index (SI) was calculated as the ratio of CC_50_ for cell growth to EC_50_ (CC_50_/EC_50_)” [65, 67].

### 2.8. Thin-Layer Chromatography (TLC) and Phytochemical Analysis

Phytochemical analysis by means of TLC was carried out for all crude extracts, as previously described by Panda et al. [65]. The TLC plate (dimensions 2.5 × 7.5 cm, coated with silica gel 60 F254, Merck, Germany) was developed with methanol : dichloromethane (95 : 5, v/v) at ambient temperature (approximately 20°C) and dried in an oven at 90°C for 5 minutes to evaporate the solvent. The plate was visualized under ultraviolet (UV) light at 254 and 360 nm. Later, the same plate was used for visualization of the spots by spraying with 5% sulphuric acid in ethanol, followed by heating at 100°C for 5 minutes. Phytochemicals were tentatively identified by comparison of Rf values and spot colours with literature data.

## 3. Results

### 3.1. Antimicrobial Testing

Since there is a renewed interest in the search for plant-based medicines using traditional knowledge (Table 1), we tested eighteen crude ethanolic/methanolic plant extracts against a panel of microbes: two Gram-positive bacteria (*S. aureus* and *L. innocua*), four Gram-negative bacteria (*E. coli*, *P. aeruginosa, S. enterica*, and *S. sonnei*), and one fungal species: *Candida albicans.* Out of these 18 plant extracts, those of *Clerodendrum serratum*, *Sapindus mukorossi, Ampelocissus tomentosa, Dicrocephala integrifolia, Boerhavia diffusa*, *Rhododendron arboreum, Justicia adhatoda*, and *Terminalia chebula* were active against a wide range of pathogens as crude extract at concentrations of 1000 *μ*g/mL (Table 2). Upon further serial dilution of active plant extracts, most of the plant extracts (especially of *S. mukorossi and D. integrifolia*) rapidly lost their activity. This might be due to low potency compounds, low concentrations of potent compounds, or coloured compounds in the extracts that interfere with spectrophotometric endpoints at high concentrations. However, some of the plant extracts, such as *A. tomentosa* and *Aleuritopteris anceps*, displayed noteworthy antimicrobial activity against *S. aureus* (MIC of 35 *μ*g/mL and 649 *μ*g/mL, respectively) and *P. aeruginosa* (MIC of 15 *μ*g/mL and 38 *μ*g/mL, respectively). Furthermore*, R. arboreum* and *J. adhatoda* were found to be active against *S. enterica* (MIC of 285 *μ*g/mL and 326 *μ*g/mL, respectively) (Table 3).

### 3.2. Anthelmintic Activity

**Figure 1:**
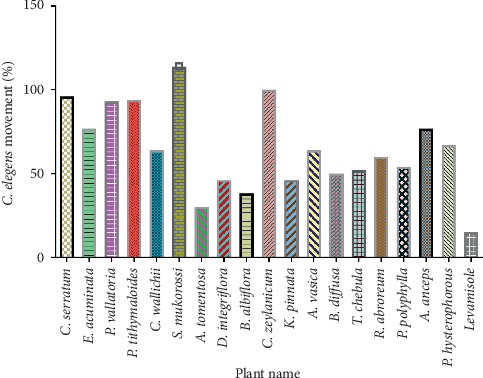
Anthelmintic activity of 18 ethnobotanically selected plants of Nepal.

We already reported the antiprotozoal activities of crude extracts of select medicinal plants of Nepal and found three active species: *Phragmites vallatoria*, *Ampelocissus tomentosa* (for which no antiprotozoal activity had been reported previously), and *Terminalia chebula* [63]. Moreover, nonselective antileishmanial activity was shown for *P. polyphylla*, *K. pinnata*, and *Terminalia chebula* [63]. Encouraged by these findings, and also by the traditional use of the plants as anthelmintics [63, 68], we tested the effects of these plant extracts on the motility of the L4 stage of *C*. *elegans.* We used this nonparasitic free-living nematode and model organism to test anthelmintic activity due to its low cost and easy availability of mutant and transgenic strains for functional genomics and proteomics studies as well as to investigate key signaling events and drug interactions and its amenability to laboratory culture methods. Furthermore, *C. elegans* is taxonomically closely related to the suborder Strongylida, which comprises several economically important animal parasites, such as *Haemonchus contortus* and *Strongyloides stercoralis,* that cause both human and animal disease [69]. Drug screening in the natural hosts for every helminthic disease is almost impossible and a costly process. So, based on the hypothesis that plant extracts affecting *C. elegans* may also have effects on other (including parasitic) nematodes, we screened our plant extracts on the surrogate parasitic nematode *C. elegans*. To monitor worm viability, we used the WMicrotracker, a device that monitors the movement of helminths in real time using infrared light beams. The advantage of using the WMicrotracker over other methods is that it enables screening of a large number of compounds in a fully automated way that is easy, fast, label-free, and highly reproducible. Interestingly, four plants were found to be active out of 18 plants tested: *A. tomentosa, B. albiflora, K. pinnata*, *and D. integrifolia.* These four plants significantly reduced *C. elegans* motility, comparable to the positive control (>50%) **(**Figure 1**)**. Moreover, plants such as *B. diffusa, P. polyphylla*, and *T. chebula* showed moderate inhibition using the WMicrotracker, but under the microscope, these extracts showed clear paralytic activity. The average inhibition was lower; however, very good activity was seen during the last four hours of WMicrotracker movement recordings, revealing that many extracts appear to have a slow onset of action.

### 3.3. Antiviral Testing

All of the plant extracts (listed in Table 1) were tested against three different viruses i.e., yellow fever, chikungunya virus (CHIKV**),** and enterovirus 71. One-third of the plants were active against these viruses. Among them, *K. pinnata* (EC_50_ = 6.1 *μ*g/mL, SI = 4.29)*, A. tomentosa* (EC_50_ = 7.79 *μ*g/mL, SI = 4.69), and *P. polyphylla* (EC_50_ = 8.74 *μ*g/mL, SI = 1.75) were active against CHIKV, *C. serratum* was active against yellow fever virus, with EC_50_ of 15.9 *μ*g/mL, and *T. chebula* (EC_50_ = 10.6 *μ*g/mL, SI = 5.94) was active against enterovirus 71 (Table 4).

### 3.4. TLC and Preliminary Phytochemical Screening

All the plant extracts were subjected to TLC, to generate fingerprints. Using methanol : dichloromethane (95 : 5, v/v) as mobile phase, numerous absorbing bands were observed under UV light (254 nm and 360 nm). Furthermore, after exposing the plates to 5% sulphuric acid in ethanol, spots were visualized, and the reaction products were compared under UV light. Interestingly, with or without derivatization, several extracts show similar fingerprints (observed colour as well as Rf), signifying the presence of similar chemical classes. Most of the plant extracts exposed to 254 nm UV light show orange, yellow, pink, green, or blue fluorescence, confirming the presence of flavonoids and polyphenols (Supplementary Figure 2). Select plant extracts such as *A. anceps, A. tomentosa,* and *B. albiflora* show similar Rf values and band patterns (blue or green fluorescence at 360 nm, converted to yellow, blue, or blue-green fluorescence after exposure to sulphuric acid) confirming the presence of coumarins (Supplementary Table 1). *C. zeylanicum* extract forms dark zones under UV 254 that disappear at 360 nm, suggesting sesquiterpene lactones. Several plant extracts such as *C. zeylanicum, D. integrifolia, E. acuminate, J. adhatoda, K. pinnata,* and *S. mukorossi* did not show any bands under UV initially but form clear bands of blue, light green, or brown colour after exposure to sulphuric acid, confirming the presence of saponins. Three plant extracts: *C. wallichii, J. adhatoda,* and *P. polyphylla,* show blue colour fluorescence turning to green or gray after exposure to sulphuric acid, confirming the presence of alkaloids. Only one extract, *R. arboreum*, shows the presence of tannins, while extracts such as *B. albiflora, C. serratum, D. integrifolia,* and *P. tithymaloides* contain sterols and glycosides. The TLC fingerprints suggest the presence of several groups of phytochemical constituents, but which of these constitute the bioactive compounds responsible for the detected activity requires further study.

## 4. Discussion

### 4.1. Antimicrobial Activity

In the area of infectious disease, of the 141 small molecules approved between 1981 and 2014 as antibacterial agents, 82, or 58%, originated from natural sources [6]. Thus, there is a huge potential for discovering new compounds with anti-infective properties from natural sources. Some of our findings are corroborated by Tantry et al. [70], who isolated oleanadien-3*β*-ethan-3-oate from *Rhododendron* species, and this compound inhibits *Salmonella typhi* (MTCC 53) (inhibition zone of 18.6 mm) at a concentration 0.1 *μ*L/mL. Similarly, Pradhan et al. [71] observed an MIC of 78.12 *μ*g/mL for a methanolic extract of *J. adhatoda* against *S. enteritidis*, in agreement with our findings. In our study, we could not find noteworthy antimicrobial activity of *K. pinnata* and *Dichrocephala integrifolia.* In contrast, Tatsimo et al. [72] investigated the antimicrobial activity of *Bryophyllum pinnatum* (synonym *K. pinnata*) on a wide panel of bacteria and yeast and found noteworthy antimicrobial activity in a crude methanolic extract, with MICs ranging from 32 to 512 *μ*g/mL. Likewise, earlier researchers have shown inhibitory effects of *D. integrifolia* against *S. aureus*, *E. coli*, and *S. typhi* in a dose-dependent manner from all crude extracts, mostly in nonpolar solvents except ethanol [73]. Several factors may explain these discrepancies: differences in the plant part or variety, soil or climate, collection or postharvest treatment, extraction procedure, etc. Furthermore, in our study, we only found moderate activity of *Clerodendrum serratum* against *S. enterica* (MIC of 882 *μ*g/mL). A large number chemical constituents (∼283 compounds) have been reported from the genus *Clerodendrum* (monoterpenes and derivatives, sesquiterpenes, diterpenoids, triterpenoids, flavonoids and flavonoid glycosides, phenylethanoid glycosides, steroids and steroid glycosides, cyclohexylethanoids, anthraquinones, and cyanogenic glycosides), showing a multitude of biological activities [74]. Arokiyaraj and colleagues measured the antimicrobial activity of *Clerodendrum siphonanthus* against *Klebsiella pneumoniae*, *Proteus mirabilis, S. typhi, S. aureus*, *E. coli,* and *Bacillus subtilis* and observed inhibition zones of 30, 16, 16, 12, 11.5, and 10 mm, respectively [75].

The present study finds broad-spectrum antimicrobial activity (extracts effective against a panel of test bacteria) with plants such as *Ampelocissus tomentosa, Boerhavia diffusa, Dichrocephala integrifolia, Pedilanthus tithymaloides, Paris polyphylla*, and *Sapindus mukorossi*. Indeed, plants such as *Boerhavia diffusa* [76], *Dichrocephala integrifolia* [73]*, Pedilanthus tithymaloides* [77], *Paris polyphylla* [78, 79], and *Sapindus mukorossi* [80, 81] were previously reported for their antimicrobial properties. However, we report here for the first time that the methanolic extract of *Ampelocissus tomentosa* root is effective against all tested pathogens: Gram-positives, Gram-negatives, and *Candida albicans.* Similarly, plants such as *Aleuritopteris anceps*, *Boenninghausenia albiflora*, *Cynoglossum zeylanicum*, *Clerodendrum serratum*, *Rhododendron arboretum*, and *Terminalia chebula* were found to show narrow-spectrum activity towards either Gram-negatives or Gram-positives. Plants such as *Boenninghausenia albiflora* [56, 82], *Clerodendrum serratum* [83, 84], *Rhododendron arboreum* [85, 86], and *Terminalia chebula* [34, 76, 87] were already reported as having antimicrobial activity. To the best of our knowledge, *Aleuritopteris anceps* and *Cynoglossum zeylanicum* are shown for the first time as active against bacteria. Three plants, that is, *Ampelocissus tomentosa*, *Dichrocephala integrifolia*, and *Paris polyphylla*, were found to inhibit significantly the growth of *Candida albicans*.

Amongst our 18 plants, the family Asteraceae is represented the most, with three plants. Despite the discovery of numerous secondary metabolites in Asteraceae, this family has received little attention in ethnopharmacological research, resulting in few systematic explorations and few commercialized products [88]. However, in the present study, one Asteraceae plant: *Cirsium wallichii,* did not show antimicrobial property against any tested microbe when we fix our cutoff at 50% inhibition. In addition, two other plants do not show antimicrobial activity: *Kalanchoe pinnata* (Crassulaceae) and *Phragmites vallatoria* (Poaceae).

### 4.2. Antiviral Activity

Phytomedicines have been an integral part of the traditional health care system in most parts of the world for thousands of years [89]. Plants contain thousands of secondary metabolites that have a broad range of bioactivities. Hence, it is interesting to test antiviral properties of plant extracts that are active against infectious diseases. For the plants we selected, we did not find any prior reports on activity against yellow fever, chikungunya, or enterovirus. Nonetheless, from a member of the same genus (*Kalanchoe gracilis),* compounds such as ferulic acid, quercetin, and kaempferol have already been isolated, which exhibited antiviral effects against enterovirus 71 (EV71) and coxsackievirus A16 (CVA16).

Chikungunya virus (CHIKV) is one of the re-emerging vector-borne viral diseases and considered as a neglected tropical disease, mainly because the affected regions are Africa and Southeast Asia. The recent outbreak of CHIKV in Central and South America also raised the possibility of massive epidemics linked to the worldwide proliferation of the mosquito vectors that transmit this disease. Moreover, there are no currently approved vaccines or antiviral treatments available yet for the prevention of CHIKV infection; it is therefore imperative to look for new bioactive molecules, and we found that several plants (*Kalanchoe pinnata, Ampelocissus tomentosa*, and *Paris polyphylla*) show activity against CHIKV (Table 4). Taking into account the IC_50_ values for inhibition of CHIKV replication and the SI value, further research is warranted on the structural elucidation of the active constituents. To identify novel inhibitors of CHIKV replication, researchers have selected Euphorbiaceae species, and upon bioassay-guided purification, they have isolated daphnane- and tigliane-type esters with anti-CHIKV activities. Euphorbia species are characterized by their ability to produce diterpenoids belonging to the tigliane, ingenane, and jatrophane chemical classes, along with other types of diterpene esters. In our study, we also found promising results of *P. tithymaloides* (Euphorbiaceae species) extracts against CHIKV; however, its antiviral action was not selective. The moderate cytotoxicity might be due to the presence of pedilstatin or eurphorbol, which have already been established as irritants and carcinogens. Likewise, a recently published manuscript by Shrestha et al. [90] reported that *Paris polyphylla* was the second most widely used plant in Eastern Nepal (use-value = 0.96, fidelity level = 77.42) to treat a wide range of ailments (Table 1). In earlier published work, we also found the promising activity of *P. polyphylla* on Leishmania; however, its action was nonselective due to its toxic effect on the MRC-5 cell line [63]. We also found activity from these plants against *Candida* and CHIKV. However, due to its toxic nature, caution is necessary since deleterious health consequences may occur if high doses are administered for a long time.

Similarly, another important viral disease (yellow fever), transmitted by mosquito bites, causes sporadic outbreaks with a case fatality rate of 15–50%, with no known cure. Though effective vaccination is available against this disease, due to inadequate coverage of vaccination in African regions and threats of re-emergence in its endemic habitat, new drugs are urgently needed. In our study, we have found an interesting activity of *C. serratum* against the yellow fever virus for the first time. Though anticonvulsant, antidiabetic, and antimalarial activity had already been reported on *C. serratum* [45], no one examined its activity against the yellow fever virus thus far. The development of new anti-yellow fever drugs from bioactive compounds obtained from these plants and the topical use of bioactive compounds present in *C. serratum* such as lupeol and pellitorine might be explored for the prevention of virus infection through the bite of the mosquito vectors.

Only one plant extract (*T. chebula*) was found to be active against enterovirus. EV71 infections can cause mild hand, foot, and mouth disease but also lead (more rarely) to severe fatal neurological complications. The anti-enterovirus activity of *T. chebula* could be due to the presence of hydrolyzable tannin (chebulagic acid), since Yang et al. [91] showed that chebulagic acid treatment reduced the viral cytopathic effect on rhabdomyosarcoma cells with an IC_50_ of 12.5 *μ*g/mL. Furthermore, in mice challenged with a lethal dose of EV71, treatment with chebulagic acid substantially reduced mouse mortality and relieved clinical symptoms through the inhibition of viral replication. *Dichrocephala integrifolia* root and flower extracts are effective in the control of human herpes simplex virus [92]. Another study conducted by Ojha et al. [42] isolated (using bioassay-guided purification) luteolin, which was active against herpes simplex virus type 2 (HSV-2).

### 4.3. Anthelmintic Activity

A search for new antiparasitic drugs has been underway over the past several decades. However, despite the abundant literature, more work is needed to yield potent, commercially available drugs based on natural products [93]. To the best of our knowledge, no anthelmintic activity has been reported previously from *A. tomentosa* and *B. albiflora*; however, insecticidal compounds coumarin, murraxocin, jayantinin, and murralongin have already been isolated from *B. albiflora* [55, 94]. Consistent with our study, Takaishi et al. [95] have characterized different isoforms of coumarin from the plant *Ageratum conyzoides* via bioassay-guided purification, and they were found to be active against a phytopathogenic nematode: *Bursaphelenchus xylophilus.* Furthermore, in a later study, the same research group has also chemically synthesized different derivatives of coumarin and found 5-ethoxycoumarin having the highest nematicidal activity, which was comparatively harmless against both the brine shrimps, *Artemia Salina,* and the Japanese killifish, *Oryzias latipes* [69, 95].

Furthermore, Wang et al. [68] investigated the anthelmintic activity from *Paris polyphylla* and found two steroidal saponins: dioscin and polyphyllin D, obtained from its methanolic extract, that exhibited significant activity against *Dactylogyrus intermedius* with EC_50_ values of 0.44 and 0.70 mg/L, respectively. These were more potent than the positive control, mebendazole (EC_50_ value = 1.25 mg/L).

Wabo et al. [22] evaluated ethanolic extracts of *D*. *integrifolia* on the gastrointestinal nematode *Heligmosomoides bakeri* and concluded that they contain compounds that have ovicidal and larvicidal properties. The ethanolic extract of *D. integrifolia* inhibited 98.1% and 98% of L1 and L2 larvae, respectively, of *Heligmosomoides bakeri* at 5 mg/mL [22]. The earlier findings on *P. polyphylla* and *D. integrifolia* as anthelmintic [22, 68] are also supported by our study using the model organism *C. elegans*.

Of course, plants traditionally used as anthelmintics by local healers do not necessarily show anthelmintic activity in *C. elegans*. Although activity in *C. elegans* does not correlate perfectly with activity against parasitic nematodes, it remains a useful preliminary test. Some plant extracts that do not exhibit direct anthelmintic activity may act as an irritant of the gastrointestinal tract and expel worms from the intestines that way.

Interestingly, one plant extract (*Sapindus mukorossi*) increased the movement of *C. elegans* (Figure 1) but the mechanisms are unclear. Many plants contain antioxidants and are known to increase lifespan, healthspan, and resistance to stress in *C. elegans* [96]. In addition, this plant did not show any cytotoxicity when tested against cell lines such as Huh, RD, and VCL (CC_50_ > 100 *μ*g/mL, Table 4), providing further evidence of nontoxicity.

The major limitation while working with crude plant extracts is that they contain complex mixtures of phytoconstituents, which may act individually or synergistically, or sometimes in an antagonistic way, resulting in varying bioactivities. Nevertheless, screening anthelmintic activity of crude extracts will help to find new anthelmintic drug lead compounds, which is a pressing need at present as there are only a limited number of anthelmintic drug classes available, and parasitic diseases have major socioeconomic consequences for humans as well as for livestock. Furthermore, the excessive use of commercially available anthelmintics (benzimidazoles, imidazothiazoles/tetrahydropyrimidines, macrocyclic lactones, and amino-acetonitrile derivatives) has led to drug resistance, and these treatments are no longer as effective as they were initially.

Efforts have been made to develop new vaccines against parasitic platyhelminthes and nematodes. Nonetheless, an efficient vaccine has not been successful because it fails to provide protective immunity. Given that no new effective anthelmintic compounds are becoming available, combined with the lack of a vaccine and emerging resistance problems against the currently available antiparasitic drugs, research into alternative sources of drugs is urgently needed. Since no human anthelmintics have been developed from plant sources so far, there is hope that new anthelmintic molecules can be found in plants that exhibit broad-spectrum activity, excellent safety, and therapeutic profiles and also display significant activity against different developmental stages of parasites [97]. Therefore, finding a broad-spectrum drug that could treat multiple parasites or multiple life stages is desirable, especially in developing countries with limited resources.

Another major limitation in our study (due to time and financial constraints) is that we could only test methanolic/ethanolic plant extracts and single plant parts. Some bioactive constituents are preferably extracted by nonpolar solvents such as chloroform or hexane, or by mild polar solvents such as ethyl acetate. In our study, we only used one polar solvent (methanol) on *D. integrifolia* and this might explain why we could not observe significant antimicrobial activity against *S. aureus* and *C. albicans*, despite this plant being used traditionally in the treatments of wound infections and other infectious ailments. The choice of an appropriate solvent is crucial for bioactivity. Solvents used in the extraction techniques and the extraction scheme change the quantity and the efficacy of the extracted phytoconstituents. For example, in the case of the famous antimalarial drug artemisinin, the hot water extract of *Artemisia annua* was ineffective in rodent mouse models infected with *P. berghei*, while the ether extract did show promising results.

Moreover, plants showing no evidence of activity in our study may be worth studying further since the plants studied were tested at 1 mg/mL, and many crude extracts may only show activity at significantly higher concentrations. Also, the activity of the extract is affected by spatiotemporal variation, the plant part taken for screening purposes, and the environmental conditions under which the plant material was harvested. These factors may also explain some of our negative results or discrepancies with earlier reports.

## 5. Conclusions

The purpose of the current study was to investigate the antiviral, antibacterial, antifungal, and anthelminthic activity of select medicinal plants of Nepal. To the best of our knowledge, this is the first systematic report on the use of plants selected based on ethnobotanical surveys (information gathered from local healers or already available in the literature) and their testing for different anti-infective bioactivities. Further work will be needed to identify the bioactive compounds from the active extracts, as well as their drug target or mode of action.

## Figures and Tables

**Table 1:** List of the plants selected for this study, their phytoconstituents, and reported traditional uses.

Name of the plant	Family	Voucher specimen	Indigenous uses	Pharmacological/phytochemical studies (literature review)
*Justicia adhatoda* L.	Acanthaceae	10027	Relieves cough, fever and breathlessness. Stops bleeding and treats rheumatic pain [12, 13].	The biological action of this plant includes abortifacient, antimicrobial, and anthelmintic activity, as well as antitussive, anti-inflammatory, and insecticidal activity [14].
*Cirsium wallichii* DC.	Asteraceae	7098	Used to treat fever and nose bleeding. Stem pith is eaten to relieve burning sensation while urinating and to control stomach inflammation [15].	The plant possesses promising antibacterial and antifungal activity against a panel of pathogens [16]. Well-known phytoconstituents are O-acetyljacoline, uteolin-7-O-glucoside, and vicenin-2 [17].
*Dichrocephala integrifolia* (L.f.) Kuntze	Asteraceae	1123	Treatment of cuts, wounds, malarial fever, and amoebiasis [18]. It is also put into the nose to treat sinusitis and migraine [19].	Diterpene lactones, sesquiterpene lactones, sterols, dicaffeoylquinic acid, and flavonoids have already been isolated from this plant [20]. Recently, anxiolytic and sedative properties of *D. integrifolia* were demonstrated in a mouse model [21]. Anthelmintic activity was observed in crude ethanolic extract of *D. integrifolia* against gastrointestinal nematode parasites of mice (*Heligmosomoides bakeri*) [22].
*Parthenium hysterophorous* (Nutt.) Torr. & A.Gray	Asteraceae	0613	Gastritis problems.	A dichloromethane extract from roots showed a MIC of 15.6 *μ*g/mL against *Helicobacter pylori* [23]. Parthenin, an *α*-methylene-*γ*-lactone sesquiterpene, has already been isolated from *P. hysterophorus,* which has shown antiamoebic activity. This activity was tested *in vitro* against axenic and polyxenic cultures of *Entamoeba histolytica* and was found to be comparable to that of the standard drug metronidazole [24].
*Cynoglossum zeylanicum* (Vahl) Brand	Boraginaceae	6053	Used for treating indigestion. Juice of the plant is applied to treat cuts and wounds [25].	Well studied for anti-inflammatory [26], antifertility [27], and antitumor activity [28] and hepatoprotective effects [29].
*Ehretia acuminata* R.Br.	Boraginaceae	4680	Local healers use it for diarrhoea treatment [30].	The plant was found to inhibit the growth of *Helicobacter pylori* [31]. In our previous study, we found it possesses antileishmanial and antimalarial activity [14].
*Terminalia chebula* Retz.	Combretaceae	599	Treatment of bleeding piles, ulcers, and gout [32].	The plant has been demonstrated to possess multiple pharmacological and medicinal activities, such as antioxidant, antimicrobial [33], antidiabetic, hepatoprotective, anti-inflammatory, antimutagenic, antiproliferative, radioprotective, cardioprotective, antiarthritic, gastrointestinal motility, and wound-healing activity [34].
*Kalanchoe pinnata* (Lam.) Pers.	Crassulaceae	2241	Treatment of kidney stones and ear infections. Juice of fresh leaves used in treatment of jaundice [35].	Literature suggests immunosuppressive effects. Also, it inhibits disease progression in *L. amazonensis*-infected individuals [36]. Furthermore, antibacterial (respiratory tract infection), antiparasitic, antidepressant, anticancer, wound-healing, anti-insecticidal, antiallergic, anti-inflammatory, and antidiabetes activity in this plant has already been reported [37, 38].
*Rhododendron arboreum* Sm.	Ericaceae	9450094	Treatment of diabetes, diarrhoea, and dysentery [39, 40].	Hyperin present in the flowers of *R. arboreum* exhibited dose-dependent and significant (*P* < 0.05–0.001) antidiarrhoeal potential in castor oil and magnesium sulphate-induced diarrhoea [41]. Ethanolic fraction from *R. arboreum* produced a significant (*P* < 0.0001) reduction in blood glucose (73.6%) at a dose of 200 mg/kg in diabetic rats [41].
*Pedilanthus tithymaloides* (L.) Poit.	Euphorbiaceae	1012	Traditionally used to reduce pain in joints.	Constituents isolated from *P. tithymaloides* (luteolin) had potent antiviral activity against wild-type and clinical isolates of HSV-2 (EC_50_ 48.5–52.6 and 22.4–27.5 *μ*g/mL, respectively) [42]. Various poly-O-acylated jatrophane diterpenoids showed antiplasmodial activity *in vitro* with IC_50_ values of 3.4–4.4 *μ*g/mL and confirmed *in vivo* showing 76% suppression in *Plasmodium berghei*-infected mice [43].
*Clerodendrum serratum* (L.) Moon	Lamiaceae	3292	Leaf juice used in treatment of malarial fever and relief from pain and eye inflammation [44].	Bioactive compounds such as icosahydropicenic acid and ursolic acid have been isolated from the root of *C. serratum,* and they have been claimed to have antiallergic and hepatoprotective activity [45]. Leaf paste is used to cure septicemia and has antimicrobial property [45].
*Paris polyphylla* Sm.	Melanthiaceae	9160305	Gastric and menstrual problems, to remove worms, and to treat cough, fever, and high blood pressure [46].	*P. polyphylla* diosgenin-type saponins revealed antileishmanial activity (IC_50_ 1.6 *μ*g/mL) but without cytotoxicity evaluation [47]. Formosanin C and polyphyllin VII identified from *P. polyphylla* were significantly effective against *Dactylogyrus intermedius* with EC_50_ values of 0.6 and 1.2 mg/L, respectively, justifying its use in traditional medicine as an anthelmintic plant [48]. Treatment of MCF-7 and MDA-MB-231 cells with polyphyllin D resulted in the inhibition of viability and induction of apoptosis in a dose-dependent manner, with an IC_50_ of 5 *μ*M and 2.5 *μ*M, respectively, after 48 hours of incubation [49].
*Boerhavia diffusa* L.	Nyctaginaceae	2681063	Whole plant paste used for healing wounds and treating swelling, back pain [50].	A total of 180 compounds from plants of the *Boerhavia* genus were isolated, of which *B. diffusa* alone contributed around 131 compounds showing vital pharmaceutical activities such as anticancer, anti-inflammatory, antioxidant, and immunomodulatory [38].
*Phragmites vallatoria* (Pluk. ex L.) Veldkamp	Poaceae	8730	Used for cuts and wounds.	Ethanol leaf extract of *Phragmites vallatoria* promotes wound healing within 11 days in rats with streptozocin-induced diabetes [51].
*Aleuritopteris anceps* (Blanf.) Panigrahi (synonym of Cheilanthes anceps Blanf.)	Pteridaceae	4163	Used in external cuts and wounds and for the treatment of ulcers, stomach problem and dysentery.	The water extract of *Cheilanthes farinosa* has shown antiproliferative and apoptotic activity in human liver cancer cells and is not deleterious for a noncancerous macrophage cell line [52]. Six flavonoid compounds (kumatakenin 5-*O*-*β*-glucoside, kaempferol-7-methyl ether, rhamnocitrin 3-*O*-*β*-glucoside, and kaempferol 3-*O*-*β*-glucoside) were isolated and identified from the ethyl acetate-soluble fraction of this plant, which has shown anti-inflammatory and antiadipogenic activities. From the *in vivo* study in rats, the crude methanol extract and phenolic fraction showed plasma triglyceride-lowering activity, as well as a reduction of weight of adipose tissue in high-fat-diet-induced obese rats [53].
*Boenninghausenia albiflora* (Hook.) Rchb. ex Meisn.	Rutaceae	86379	Taken orally as a pain killer by women to relieve stomach pain after delivery. This plant is commonly used against fever [54].	Coumarin isolated from leaves of *Boenninghausenia albiflora* showed insecticidal activity at concentrations varying from 1% to 5% w/v [55]. Recently reported as having strong antibacterial properties with toxicity in Wistar rats [56].
*Sapindus mukorossi* Gaertn.	Sapindaceae	6734	Removing dandruff and for treatment of joint pain [57].	Daily oral administration of *Sapindus mukorossi* fruit extract (250 and 500 mg/kg body weight) and glybenclamide for 20 days showed beneficial effects on blood glucose levels and lipid levels [58]. Constituents from *Sapindus* (sapindoside G and 4″,4″‴-O-diacetylmukurozioside) exhibited inhibitory effects against A549 human lung adenocarcinoma cells: 69.2–83.3% inhibition at a concentration of 100 *μ*g/mL [33].
*Ampelocissus tomentosa* (B.Heyne & Roth) Planch.	Vitaceae	308	Root paste is applied externally to cuts and wounds and muscular swelling until they cure [59].	Grape extracts were found to have anti-inflammatory activity [60]. Polyphenolic fruit extracts contain catechin, epicatechin, delphinidin, malvidin, myricetin, cyanidin, procyanidin B1 and B2, peonidin, petunidin, resveratrol, and quercetin [61, 62].

**Table 2:** Antibacterial and antifungal activity of the extracts of 18 ethnobotanically selected plants of Nepal (%, relative inhibition compared to the solvent, OD at 620 nm).

Plant name	Solvent	Part used	*E. coli*	*P. aeruginosa*	*S. enterica*	*S. sonnei*	*S. aureus*	*L. innocua*	*C. albicans*
*Aleuritopteris anceps*	MT	L	49	99	7	43	75	40	33
*Ampelocissus tomentosa*	MT	R	100	100	14	93	100	100	62
*Boenninghausenia albiflora*	MT	B	61	95	78	40	0	46	42
*Boerhavia diffusa*	MT	WP	100	100	93	95	10	100	31
*Cirsium wallichii*	ET	L	19	7	20	24	0	45	19
*Clerodendrum serratum*	ET	WP	18	4	93	6	0	44	26
*Cynoglossum zeylanicum*	MT	WP	56	18	70	40	17	63	31
*Dichrocephala integrifolia*	MT	L	100	100	64	34	0	100	23
*Ehretia acuminata*	ET	L	15	38	32	59	14	17	29
*Justicia adhatoda*	MT	R	61	62	93	55	0	78	22
*Kalanchoe pinnata*	ET	L	17	42	43	19	39	15	39
*Paris polyphylla*	MT	L	57	100	67	47	80	65	99
*Parthenium hysterophorus*	MT	Rh	34	2	94	ND	10	53	29
*Pedilanthus tithymaloides*	ET	L	74	100	58	23	2	67	26
*Phragmites vallatoria*	ET	WP	42	10	10	36	0	34	27
*Rhododendron arboreum*	MT	L	33	18	100	9	63	34	40
*Sapindus mukorossi*	ET	Fr	100	100	97	90	25	100	22
*Terminalia chebula*	MT	L	48	38	53	27	75	43	38
Ciprofloxacin			97	—	—	100	100	98	—
Miconazole			—	—	—	—	—	—	92

ET, ethanol; MT, methanol; L, leaves; R, root; B, bark; Fr, fruit; WP, whole plant; Rh, rhizome; ND, not determined.

**Table 3:** Minimum inhibitory concentration of active plant extracts of ethnobotanically selected plants of Nepal.

Plant name	Bacteria tested (IC_50_, *μ*g/mL)					
	*E. coli*	*S. aureus*	*P. aeruginosa*	*L. innocua*	*S. enterica*	*S. sonnei*
*Aleuritopteris anceps*	—	649	38	—	—	—
*Ampelocissus tomentosa*	196	35	15	—	—	627
*Boerhavia diffusa*	—	—	669	592	—	—
*Clerodendrum serratum*	—	—	—	—	882	—
*Dichrocephala integrifolia*	—	—	651	97	—	—
*Justicia adhatoda*	—	—	—	—	326	—
*Rhododendron arboreum*	—	—	—	—	285	—
*Sapindus mukorossi*	—	—	619	—	—	—
*Terminalia chebula*	—	600	—	—	—	—

Note: for some of the plant extracts tested against different bacteria, data are not shown due to sudden loss of activity upon further dilution. —, not determined.

**Table 4:** Antiviral activities of 18 ethnobotanically selected plants of Nepal.

Plant name	Part used	Yellow fever virus			Chikungunya virus			Enterovirus 71		
		EC_50_	CC_50_ (huh)	SI	IC_50_	CC_50_ (VCL)	SI	EC_50_	CC_50_ (RD)	SI
*Aleuritopteris anceps*	L	—	—	—	>100	>100	—	90.8	100	>1.1
*Ampelocissus tomentosa*	R	—	—	—	7.79	36.6	4.69	—	—	—
*Boenninghausenia albiflora*	B	36.1	>100	2.77	—	54.7	—	>100	>100	—
*Boerhavia diffusa*	V	>100	>100	—	>100	>100	—	>100	>100	—
*Cirsium wallichii*	L	>100	>100	—	>100	>100	—	>100	>100	—
*Clerodendrum serratum*	WP	15.9	48.9	3.07	>100	>100	—	>100	>100	—
*Cynoglossum zeylanicum*	WP	>100	>100	—	>100	>100	—	56.6	>100	>1.76
*Dichrocephala integrifolia*	L	>100	>100	—	>100	>100	—	>100	>100	—
*Ehretia acuminata*	L	>100	>100	—	>100	>100	—	65.7	100	>1.52
*Justicia adhatoda*	R	>100	>100	—	47.7	>100	—	>100	>100	—
*Kalanchoe pinnata*	L	—	—	—	6.1	26.2	4.29	—	15.5	—
*Paris polyphylla*	L	>100	>100	—	8.74	15.3	1.75	>100	>100	—
*Parthenium hysterophorus*	Rh	>100	>100	—	>100	>100	—	>100	>100	—
*Pedilanthus tithymaloides*	L	56.5	>100	>1.77	10.5	20	1.9	>100	>100	—
*Phragmites vallatoria*	WP	>100	>100	—	>100	>100	—	>100	>100	—
*Rhododendron arboreum*	L	>100	>100	—	>100	>100	—	>100	>100	—
*Sapindus mukorossi*	Fr	>100	>100	—	40.5	>100	>2.47	>100	>100	—
*Terminalia chebula*	L	<0.8		—	>100	>100	—	10.6	63.1	5.94

All concentrations mentioned are in *μ*g/mL. Huh, human hepatoma cell line; RD, rhabdosarcoma cell line; VC, Vero cell line; EC_50_ = 50% effective concentration (concentration at which 50% inhibition of virus replication is observed); CC_50_ = 50% cytostatic/cytotoxic concentration (concentration at which 50% adverse effect is observed on mammalian host cells used for antiviral assay). SI  =  selectivity index (CC_50_/EC_50_); —, data absent; L, leaves; R, root; B, bark; Fr, fruit; WP, whole plant; Rh, rhizome.

## Data Availability

All data generated or analysed during this study are included in this published article and its supplementary material.

## Abbreviations

ATCC: American Type Culture Collection, Manassas, Virginia, USA
CC_50-:_: 50% cytostatic/cytotoxic concentration
CHIKV: Chikungunya virus
CPE: Cytopathic effect
DMSO: Dimethyl sulphoxide
EC_50_: 50% effective concentration
EV71: Enterovirus 71
HSV-2: Herpes simplex virus type 2
Huh: Human hepatoma cell line
MHA: Mueller–Hinton agar
MIC_50_: Minimum inhibitory concentration required to inhibit the growth of organisms by 50%
MTS: (3–[4,5,dimethylthiazol–2–yl]–5–[3–carboxymethoxy–phenyl]–2–[4– sulfophenyl]–2H–tetrazolium, inner salt
NGM: Nematode growth medium
OD: Optical density
RD: Rhabdosarcoma cell line
SI: Selectivity index
TI: Therapeutic index
VCL: Vero cell line
VC: Virus controls
YPD: Yeast extract peptone dextrose medium.

## Supplementary Materials

Supplementary Materials Figure 1: TLC of crude extracts exposed to UV at 360 nm; mobile phase: methanol : dichloromethane (95 : 5, v/v). Figure 2: TLC of crude extracts exposed to UV at 254 nm; mobile phase: methanol : dichloromethane (95 : 5, v/v). Figure 3: TLC of crude extracts after spraying with 5% sulphuric acid. Figure 4: TLC of crude extracts exposed to UV at 360 nm after spraying with 5% sulphuric acid. Figure 5: TLC of crude extracts exposed to UV at 254 nm after spraying with 5% sulphuric acid. Table 1: interpretation of TLC results of crude extracts.
