# Supplementary material for: Antibacterial, Antifungal, Antiviral, and Anthelmintic Activities of Medicinal Plants of Nepal Selected Based on Ethnobotanical Evidence
Source: Evid Based Complement Alternat Med. 2020 Apr 22;2020:1043471. doi: 10.1155/2020/1043471 (PMC7193273; doi:10.1155/2020/1043471)
Supplement: Supplementary Materials — Figure 1: TLC of crude extracts exposed to UV at 360 nm; mobile phase: methanol : dichloromethane (95 : 5, v/v). Figure 2: TLC of crude extracts exposed to UV at 254 nm; mobile phase: methanol : dichloromethane (95 : 5, v/v). Figure 3: TLC of crude extracts after spraying with 5% sulphuric acid. Figure 4: TLC of crude extracts exposed to UV at 360 nm after spraying with 5% sulphuric acid. Figure 5: TLC of crude extracts exposed to UV at 254 nm after spraying with 5% sulphuric acid. Table 1: interpretation of TLC results of crude extracts. [file 1043471.f1.pdf]

Supplementary Material

Antibacterial, Antifungal, Antiviral and Anthelmintic Activities of medicinal Plants of Nepal selected based on Ethnobotanical Evidence

Bishnu Joshi[1,2*¥], Sujogya Kumar Panda[3*¥], Ramin Saleh Jouneghani[1], Maoxuan Liu[1], Niranjan Parajuli[4], Pieter Leyssen[5], Johan Neyts[5], Walter Luyten[3]

Supplementary Data (photographs of TLC of crude extracts)

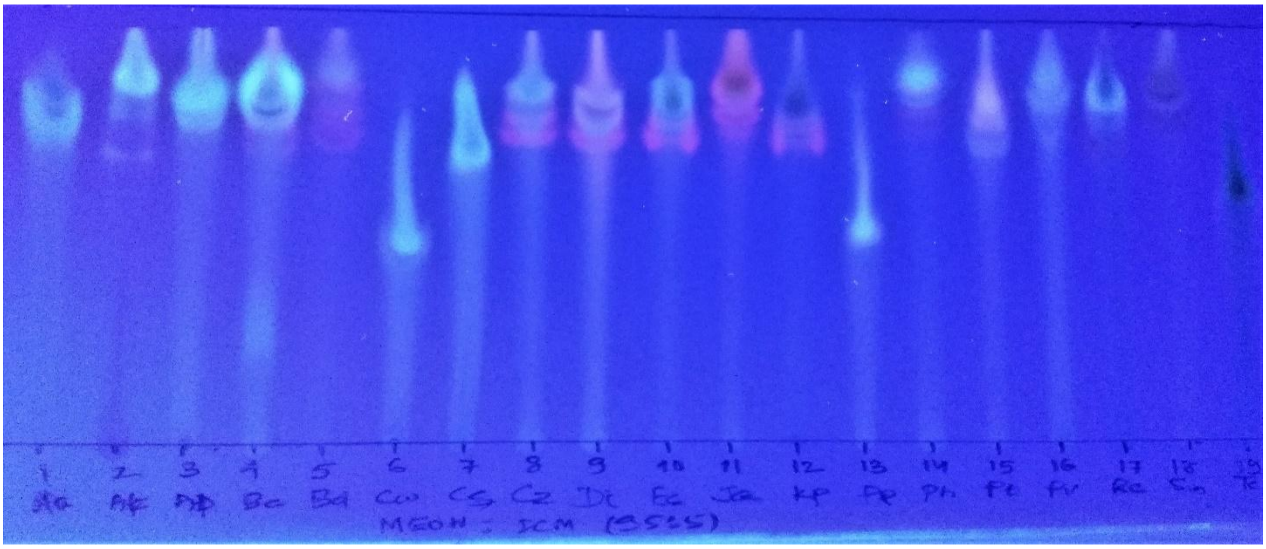

Figure 1: TLC of crude extracts exposed to UV at 360 nm; mobile phase: methanol:dichloromethane (95:5, v/v)

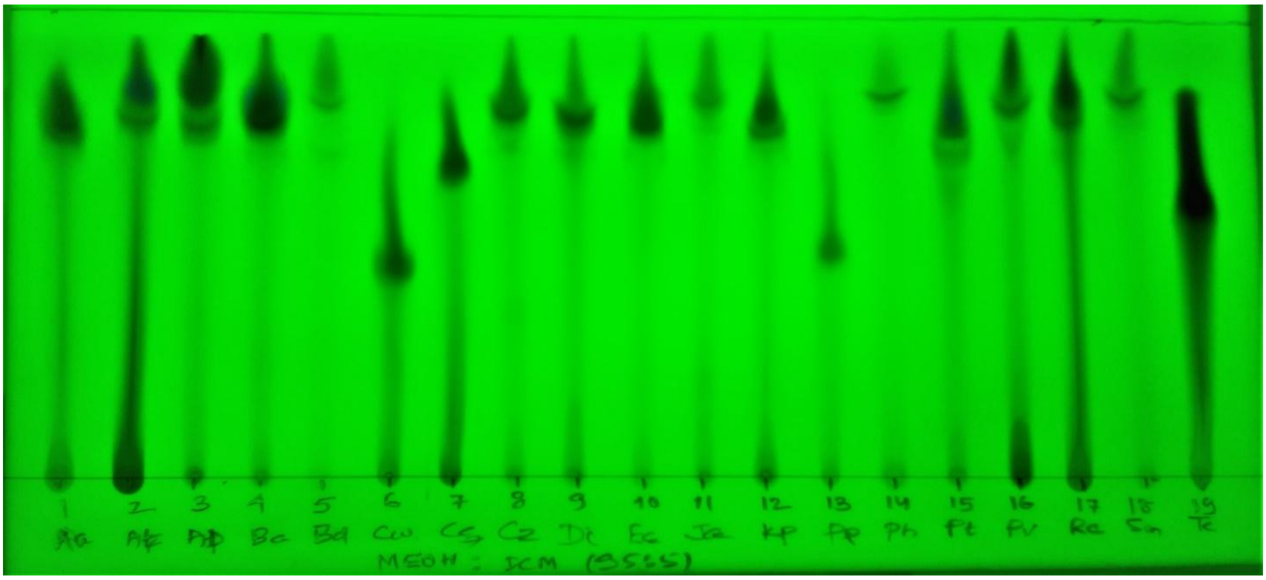

Figure 2: TLC of crude extracts exposed to UV at 254 nm; mobile phase: methanol:dichloromethane (95:5, v/v)

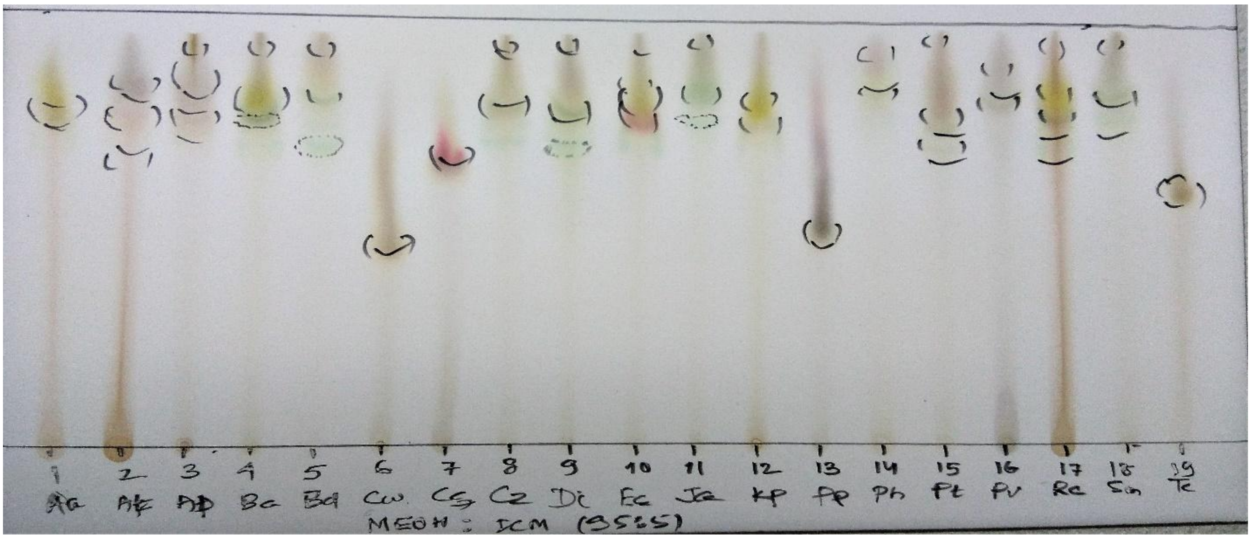

Figure 3: TLC of crude extracts after spraying with 5% sulphuric acid

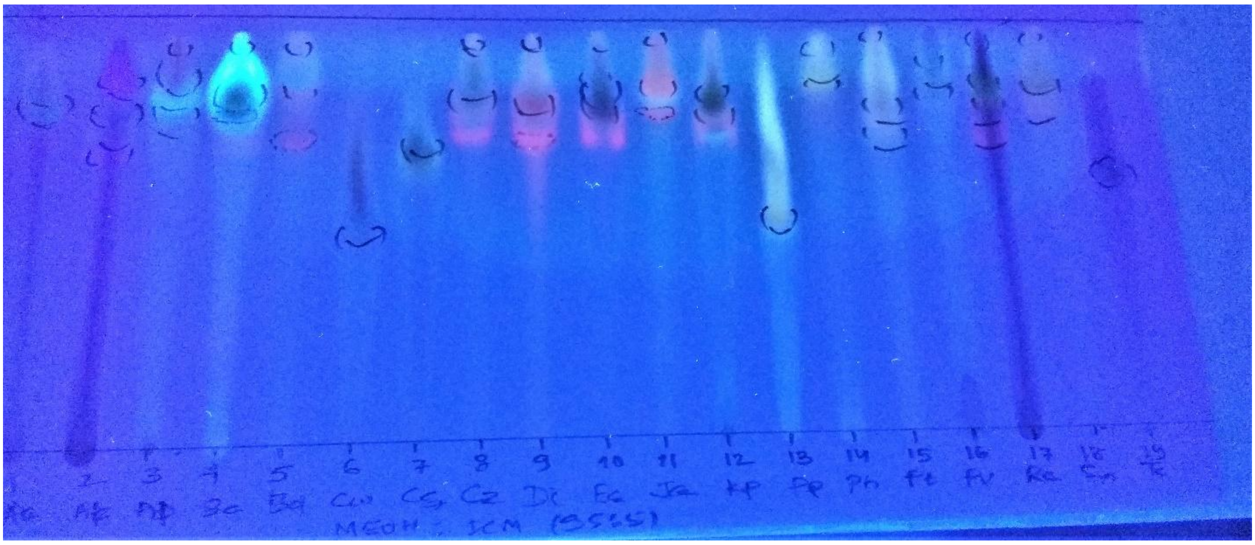

Figure 4: TLC of crude extracts exposed to UV at 360 nm after spraying with 5% sulphuric acid

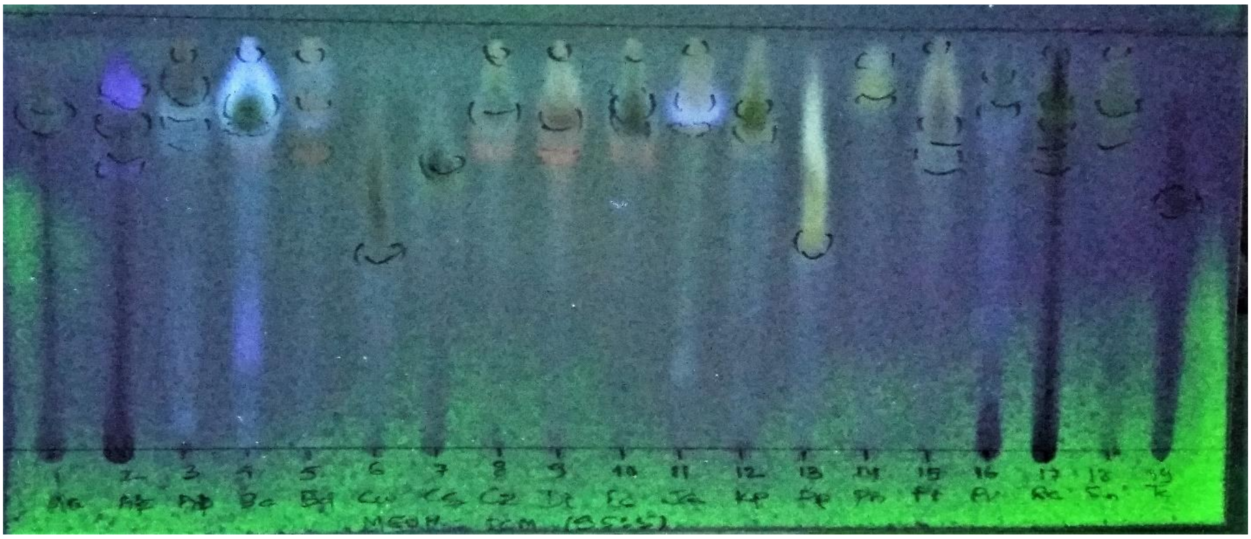

Figure 5: TLC of crude extracts exposed to UV at 254 nm after spraying with 5% sulphuric acid

Table 1: Interpretation of TLC results of crude extracts

Plant name	R_f at 254 nm	R_f at 254 nm after exposure to 5% sulphuric acid	R_f at 360 nm	R_f at 360 nm after exposure to 5% sulphuric acid	Appearance after exposure to 5% sulphuric acid	Possible phytochemical group(s)
Aleuritopteris anceps	0.8-Blue	0.8-Yellow	0.8-Brown	0.8-Yellow	0.8-Light yellow	Coumarins
	–	–	–	0.82-Pink	Brown	Flavonoids
Ampelocissus tomentosa	0.82-Blue	0.82-Blue	0.82-Blue	0.82-Blue	0.82-Gray	Coumarins
	–	–	0.75-Blue	0.75-Brown	–	Polyphenols
	–	–	–	0.77-Blue	–	–
Boenninghausenia albiflora	0.82-Blue	0.82-Blue	0.82-Blue	0.82-Blue	0.82- Gray green	Coumarins
	0.77-Light brown	0.77-Yellow	0.77-Brown	0.77-Puple	0.77-Light yellow	Flavonoids
	–	0.29-Blue	0.29-Brown	0.29-Blue	–	Sterols and glycosides
Boerhavia diffusa	0.8-Red	0.8-Red	0.8-Red	0.8-Yellow	0.8-Light green	Flavonoids or steroids
	0.69-Purple	0.69-Purple	0.69-Red	0.69-Yellow	0.69-Green	Terpenoids
Cirsium wallichii	0.47-Yellow	–	0.47-Blue	0.47-Blue	0.47-Grey	Alkaloids
Clerodendrum serratum	0.75-Light purple	–	0.66-Blue	0.66-Brown surrounded by blue	0.66-Brown	Sterols and glycosides
Cynoglossum zeylanicum	–	–	0.69-Red	0.69-Pink	0.69– Light green	Saponins
	–	–	0.77-Blue	0.77-Yellow	–	–
	0.8-Purple	0.8-Pink	–	–	0.8-Light green	Sesquiterpene lactones
	–	–	0.85-Orange	0.85-Yellow	–	Flavonoids
Dichrocephala integrifolia	0.69-Yellow	0.69-Pink	0.69-Red	0.69-Pink	0.69-Green	Saponins
	0.8-Purple	–	0.8-Blue	0.8-Brown	0.8-Light purple	Sterols and glycosides
	–	–	0.9-Orange	0.9-Yellow	–	Flavonoids

Ehretia acuminata	–	–	0.75-Red	0.75-Pink	0.75– Light green	Saponins
	–	0.8-Pink	0.8-Blue	0.8-Brown	0.8-Brown	Polyphenols
	–	–	0.82-Orange	0.82-Yellow	–	Saponins
	0.85-Blue	–	–	–	0.85-Light yellow	–
Justicia adhatoda	–	–	0.75-Red	0.75-Pink	0.75– Light green	Saponins
	–	0.8-Pink	0.8-Blue	0.8-Blue	–	Flavonoids or polyphenols
	0.85-Purple	0.85-Blue	0.85-Orange	–	0.85-Green	Alkaloids
Kalanchoe pinnata	0.77-Yellow	–	0.77-Red	0.77-Brown	–	Flavonoids or polyphenols
	–	–	–	–	0.82-Light yellow	Saponins
Paris polyphylla	0.5-Yellow	–	0.5-Blue	0.5-Yellow	0.5-Gray	Alkaloids
Parthenium hysterophorus	0.86-Light green	0.86-Yellow	0.86-Red	0.86-Brown	0.86-Light green	Flavonoids or polyphenols
	–	–	0.9-Orange	0.9-Yellow	–	Flavonoids
Pedilanthus tithymaloides	–	0.69-Blue	0.69-Pink	0.69-Red	0.69-Gray	Sterols and glycosides
	–	0.74-Blue	0.74-Purple	0.74-Brown	0.74-Light green	Polyphenols
	–	–	0.9-Orange	0.9-Yellow	–	Flavonoids
Phragmites vallatoria	–	0.8-Red	0.8-Yellow	0.8-Yellow	–	Flavonoids
	–	0.85-Yellow	0.85-Blue	0.85-Brown	0.85-Gray	Polyphenols
Rhododendron arboreum	–	0.8-Pink	0.8-Pink	0.8-Red	0.8-Light purple	Tannins
	–	0.82-Yellow	0.82-Blue	0.82-Red	0.82-Yellow	Flavonoids
	–	–	0.9-Orange	0.9-Yellow	–	Flavonoids
Sapindus mukorossi	–	0.73-Brown	0.73-Red	0.73-Pink	–	Flavonoids or polyphenols
	–	0.8-Purple	0.8-Yellow	0.8-Pink	–	Flavonoids or polyphenols
	–	–	–	–	0.85-Light green	Saponins
Terminalia chebula	0.64-Blue	–	0.64-Yellow	0.64-Blue	0.64-Gray	Coumarins
